# Shugan granule contributes to the improvement of depression‐like behaviors in chronic restraint stress‐stimulated rats by altering gut microbiota

**DOI:** 10.1111/cns.13881

**Published:** 2022-06-17

**Authors:** Junnan Li, Yannan Li, Wenzhe Duan, Zhonghui Zhao, Lixuan Yang, Wei Wei, Jingchun Li, Yang Li, Yao Yu, Baoan Dai, Rongjuan Guo

**Affiliations:** ^1^ Second Clinical Medical College Beijing University of Chinese Medicine Beijing China; ^2^ Beijing Hospital of Traditional Chinese Medicine Capital Medical University Beijing China; ^3^ Beijing Changping Hospital of Integrated Chinese and Western Medicine Beijing China; ^4^ Department of Neurology Dongfang Hospital Beijing University of Chinese Medicine Beijing China

**Keywords:** chronic restraint stress, depression‐like behaviors, gut metabolome, gut microbiota, Shugan granule

## Abstract

**Aim:**

The investigation aims to evaluate the potential effect of Shugan Granule (SGKL) on the gut, brain, and behaviors in rats exposed to chronic restraint stress (CRS).

**Methods:**

The fecal microbiota and metabolite changes were studied in rats exposed to CRS and treated with SGKL (0.1 mg/kg/day). Depressive behaviors of these rats were determined through an open‐field experiment, forced swimming test, sucrose preference, and weighing. Moreover, LPS‐stimulated microglia and CRS‐stimulated rats were treated with SGKL to investigate the regulation between SGKL and the PI3K/Akt/pathway, which is inhibited by LY294002, a PI3K inhibitor.

**Results:**

(i) SGKL improved the altered behaviors in CRS‐stimulated rats; (ii) SGKL ameliorated the CRS‐induced neuronal degeneration and tangled nerve fiber and also contributed to the recovery of intestinal barrier injury in these rats; (iii) SGKL inhibited the hippocampus elevations of TNF‐α, IL‐1β, and IL‐6 in response to CRS modeling; (iv) based on the principal coordinates analysis (PCoA), SGKL altered α‐diversity indices and shifted β‐diversity in CRS‐stimulated rats; (v) at the genus level, SGKL decreased the CRS‐enhanced abundance of *Bacteroides*; (vi) *Butyricimonas* and *Candidatus Arthromitus* were enriched in SGKL‐treated rats; (vii) altered gut microbiota and metabolites were correlated with behaviors, inflammation, and PI3K/Akt/mTOR pathway; (viii) SGKL increased the LPS‐decreased phosphorylation of the PI3K/Akt/mTOR pathway in microglia and inhibited the LPS‐induced microglial activation; (ix) PI3K/Akt/mTOR pathway inactivation reversed the SGKL effects in CRS rats.

**Conclusion:**

SGKL targets the PI3K/Akt/mTOR pathway by altering gut microbiota and metabolites, which ameliorates altered behavior and inflammation in the hippocampus.

## Graphical abstract

Gut microbiota is explicitly associated with the development of depressive disorder. However, the molecular pathogenesis associated with gut microbiota with MDD remains unclear. Shugan granule (SGKL), a Chinese patent medicine, is a potential anti‐depression drug via gut microbiota, but the molecular therapeutic mechanism of SGKL remains unknown for the depression process. Our previous study determined SGKL might contribute to alleviating depression symptoms through the PI3K/Akt/mTOR pathway. There is a lack of the investigation to demonstrate whether SGKL can target PI3K/Akt/mTOR pathway based on altered gut microbiota, leading to the improvement of depression progression. We showed a novel mechanism of gut microbiota in causing depression; gut microbiota cause changes in phosphorylation of the PI3K/Akt/mTOR pathway, which mediates microglial activation and subsequent inflammation, eventually causing the development of depression. Importantly, SGKL treatment targets the PI3K/Akt/mTOR pathway in improving depression‐like behaviour, microglia activation, and inflammation. Moreover, we concluded that *Parasutterella*, *Aeromonas*, *Ruminococcaceae_UCG_008*, *Globiocatella, Shuttleworthia*, and *Clostridium_sensu_stricto_1* were the biomarkers of depression, and *Candidatus Arthromitus* might be the therapeutic target of SGKL during improving depressive‐like behaviours. These findings suggests that SGKL may be the reliable and rational drug for the treatment in depression.

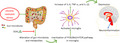

## 1 INTRODUCTION

Depression, a common illness dependent on genomic and other biological factors, is defined as a mental disorder that evokes functional impairment and represses quality of life.^1^ Depression has been considered a global public health event independent of age and socioeconomic factors.^2^ Among depression symptoms, major depressive disorder (MDD) is the most serious symptom characterized by cognitive dysfunction, affective disorder, and psychosocial impairment.^3^ According to a cross‐sectional study,^4^ patients with MDD have a high risk of developing suicidal ideation. The 12‐month prevalence of MDD is 2.3% in China.^5^ Treatment and management of MDD are challenging. For example, the pathogenesis of MDD is a complex biological network involved in neuronal remodeling, epigenetics, immune inflammation, gliocyte function, and gut microbiota,^6^, ^7^ indicating the definite mechanism of MDD has been unclear. Microglial activation in the central nervous system results from neuroinflammation, which develops into depression and associated impairments of neuroplasticity and neurogenesis.^8^, ^9^ Increasing numbers of investigations have determined that altered gut microbiota was explicitly associated with the development of depression. In the process of the gut to brain, extrinsic nociceptors may respond to altered gut microbiota during intestinal impairment, thereby resulting in the transmission of the extrinsic signal to the central nervous system (CNS).^10^ Zhang et al.^11^ found that there is a significant alteration in gut microbiota composition during CNS disorder. Interestingly, the altered gut microbiota is correlated to spontaneous brain activity and cognitive function.^12^ A previous investigation^13^ reported that gut microbiota‐correlated amino acid metabolism developed depression‐like behaviors in vivo. Han et al.^14^ suggested that treatments with Lactobacillus reuteri NK33 and Bifidobacterium adolescentis NK98 contributed to the alleviation of depression by improving the altered populations of gut microbiota and regulating NF‐κB activation. A report^15^ based on an animal model with unpredictable chronic mild stress revealed that the gut microbiota‐regulated endocannabinoid system significantly alleviated fatty acid metabolism and subsequently depression‐like behaviors. Valles‐Colomer^16^ concluded that *Dialister* and *Coprococcus* are positively related to the quality of life and depleted in treatment‐free depression, indicating the potential role of gut microbiota in depression progress. Alterations in gut microbiota are involved in pro‐inflammatory cytokine production and cell death in neurons.^17^ Thus, targeting gut microbiota will be instrumental in the improvement of neuroinflammation‐associated CNS disorder.^18^ In neuroinflammation conditions, exposure to microbiota induces inflammasome complex to be enriched in cells in CNS when cells activate and then trigger the release of pro‐inflammatory cytokines.^19^ The special mechanism suggests a possible relation between gut microbiota and microglia, one of the CNS cells, during depression progression. However, the concrete mechanism of microbiota to microglia has been not yet fully understood in MDD.

The lack of an effective therapy for this physiological and psychological disease makes MDD treatment challenging. It is estimated that a half of the patients with depression receive inadequate treatment, partly because of standard treatment resistance.^20^ Thus, a novel and effective drug is required for depression treatment. Shugan granule (SGKL) is a Chinese patent medicine that comprises angelica sinensis, radix bupleuri, rhizoma cyperi, radix paeoniae alba, rhizoma atractylodis macrocephalae, mint, poria cocos, gardenia jasminoides, cortex moutan, and licorice. SGKL functions as the inhibitor of hyperplastic disease of the breast by repressing cell proliferation and p53 expression.^21^ SGKL suppresses the population of gut mucosal serotonin‐positive cells to improve irritable bowel syndrome.^22^ Importantly, SGKL significantly evoked the reductions in CRF and ACC in the hypothalamus and alleviated depression‐like behaviors in an animal model,^23^ which suggest its potential role in depression treatment. Nevertheless, the molecular therapeutic mechanism of SGKL remains unknown for the depression process. Clinically, SGKL administration is processed orally, possibly indicating the potential role in the gut microenvironment, particularly in microbiota composition. Given the gut–brain axis effecting depression progression, we guess SGKL may play the anti‐depression role via gut microbiota‐mediated pathogenesis of MDD, which requires experimental evidence.

Our previous investigation based on network pharmacology revealed that SGKL targets the PI3K/Akt/mTOR pathway, indicating that SGKL might contribute to alleviating depression symptoms through the PI3K/Akt/mTOR pathway. The PI3K/Akt/mTOR pathway is not only a critical cellular signaling pathway involved in malignant tumor such as breast cancer,^24^ prostate cancer,^25^ acute myeloid leukemia,^26^ osteoarthritis,^27^ and ovarian cancer,^28^ but also contributes to is depression‐like behaviors and autophagy of hippocampal neurons in animal models.^29^ Tao et al.^30^ showed that PI3K/Akt/mTOR pathway activation could improve BDNF–TrkB pathway‐mediated depression‐like behaviors in rats. Notably, the mTOR pathway mediates autophagy, growth, and lipid metabolism stimulated by gut metabolism in cells and monitors gut microbiota composition through the gut barrier.^31^ A study^32^ revealed that microbiota‐derived short‐chain fatty acids elevated the production of IL‐22 in CD4+ T cells through mTOR mediation. These reports suggest that the PI3K/Akt/mTOR pathway might regulate gut metabolites in depression progress.

Thus, this investigation is based on a novel hypothesis that SGKL targets the PI3K/Akt/mTOR pathway by altering gut microbiota and metabolites, thereby contributing to the amelioration of altered behavior and neuroinflammation.

## 2 METHODS

### 2.1 Experiment 1

#### 2.1.1 Chronic restraint stress modeling

Adult male SD rats (6–8 weeks of age) weighing 180–200 g were purchased from Vital River Laboratory Animal Technology Co. Ltd (Beijing, China). The rats were randomly divided into three groups: control group (*n* = 8) containing rats not exposed to any stress; CRS group (*n* = 8) exposed to CRS; and SGKL group (*n* = 8) exposed to CRS on SGKL treatment. The rats were subjected to 6 h of restraint stress in a mineral water bottle every day at random times after 1‐week habituation to the environment. CRS modeling was performed continually for 4 weeks. After CRS modeling each day, SGKL treatment was immediately administered at the dose of 0.63 g/kg/day. The treatment was continued for 4 weeks. Behavioral tests and weighing were performed after modeling. All animal works were conducted with the approval of the Ethics Committee of China‐Japan Friendship Hospital (zryhyy21‐20‐09‐9).

### 2.2 Experiment 2

The rats were randomly divided into four groups: control group (*n* = 8) without any stress; CRS group (*n* = 8) exposed to CRS; SGKL group (*n* = 8) exposed to CRS with SGKL treatment; and SGKL+LY294002 group (*n* = 8) based on the administration of SGKL group and infused with LY294002 (25 μg in 0.5‐μl DMSO, Solarbio, Beijing, China), a PI3K inhibitor, in the hippocampus. The method of intrahippocampal infusion was performed, as described in a previous study.^33^

### 2.3 Behavioral test

We performed the sucrose preference test, open‐field test, and forced swimming test to assess the behaviors of rats. The rats were subjected to behavioral tests after modeling, and they were acclimated to text condition for 30 min before the tests. *Sucrose preference test*: The rats in cages were cultured without food and water for 24 h, after which two bottles were simultaneously placed into each cage. The rat could freely access the two bottles. We weighed the two bottles after the 10‐h test. Sucrose preference was calculated as follows: sucrose preference = [intake of sucrose/(intake of sucrose + intake of water)] × 100%. The reduction of sucrose preference mean animals was in depression. *Open‐field test (OFT)*: Open‐field test was performed in a plastic chamber (72 × 72 × 40 cm^3^) without a ceiling. The chamber was cleaned with 70% ethanol and covered with corn kernels on the base before each test. Each rat was placed at the center of the chamber and allowed to move for 4 min freely. The latency time to enter the central part, crossing and rearing behaviors of rats, was recorded by a video camera system located above the chamber. The increase in crossing counts, rearing counts, or immobile time in the corner indicates the depression. *Forced swimming test (FST)*: We performed the forced swimming test in a glass cylinder with a diameter of 25 cm and a height of 50 cm. The glass cylinder contained 35‐cm deep water at 25 ± 1°C. Each rat was forced to swim for 6 min (the first 2 min for the pre‐experiment, and the last 4 min for the formal test). The time of immobility was measured in the last 4 min. An increase in immobile time was an indication of depression.

### 2.4 Histological staining

After the last trial, we assessed pathological changes in the hippocampus or colonic tissue isolated from rats through H&E staining or Nissl staining. Each rat provided one sample of the hippocampus and one of colonic tissue. We investigated the activation of microglia in the CA1 hippocampus through IBA‐1. Before histological staining, the tissues were incubated with 4% paraformaldehyde solution for fixation and embedded in paraffin after dehydration. These samples were sectioned into 4‐μm slices for histological staining. *H&E staining* (Beyotime, Shanghai, China): CA3 hippocampus or colonic tissue sections were incubated with hematoxylin for 10 min, followed by eosin staining for 2 min. *Nissl staining* (Beyotime, Shanghai, China): samples of the CA3 hippocampus were stained with Nissl staining solution for 10 min at 37°C. We observed the pathological changes through a microscope (Olympus, Japan). *Immunofluorescence staining*: sections of CA1 hippocampus were fixed in a 4% paraformaldehyde solution overnight at 4°C after dewaxing, hydration, and antigen repair, followed by blocking with normal sheep serum (Solarbio, Beijing, China) for 1 h at room temperature and incubation with the anti‐IBA‐1 antibody (ab178680, Abcam, UK, 1/500) at 4°C overnight. An anti‐rabbit IgG (Abcam, 1/300) was added to the sections, and the samples were incubated overnight at 4°C in the dark before re‐staining of 594‐conjugated AffiniPure Donkey (Jackson, USA, 1/1000) for 7–8 min at room temperature. Using a laser confocal microscope (Leica, GER), five visual fields in each image were randomly selected to count the numbers of IBA‐1 positive cells. Then, results from the five counts were averaged. The cells with the cell body stained were the IBA‐1‐positive cells and the small fragments were excluded.

### 2.5 Culture and administration of microglia

Rat microglia (HAPI) were cultured in cell culture flasks supplemented with RPIM 1640 medium (HyClone, USA) containing 10% fetal bovine serum (FBS, Gibco, USA, v/v), 100 U/mL penicillin (Nanjing KeyGen Biotech Co. Ltd, Nanjing, China), and 100 U/mL streptomycin (Nanjing KeyGen Biotech Co. Ltd, Nanjing, China) at 37°C with 5% CO_2_. For successive administrations, the cells were seeded in a 24‐well plate at the density of 20,000 cells at 37°C with 5% CO_2_ for 24 h. *LPS stimulation* ^34^: HAPI were stimulated with lipopolysaccharide at the concentration of 1 μg/ml for 6 h. *SGKL treatment*: LPS‐stimulated cells were treated with 7.11 mg/mL of SGKL for 72 h. SGKL dose is described in Supplementary Material (Figure S1). Cells in the SGKL+LY294002 group were incubated with LY294002 (50 μM in DMSO^35^) combined with SGKL treatment after LPS stimulation. The cells were incubated with an equal volume of DMSO as the control of LPS stimulation. *Immunofluorescence staining of HAPI*: HAPI were fixed in 4% paraformaldehyde overnight at 4°C and blocked by TBS containing 0.1% Triton X‐100, and 5% BSA for 1 h with shaking at 90 rpm, followed by the steps described under section: 2.4 Histological Staining Immunofluorescence staining.

### 2.6 Western blot

Total protein in microglia was extracted using RIPA lysis buffer (Solarbio, China) and then separated using the SDS‐PAGE system (Bio‐Rad, USA) through electrophoresis. The protein was transferred to the PVDF membrane (Millipore, Germany) and incubated with anti‐PI3K (ab191606, 1/1000), anti‐AKT (ab179463, 1/10000), anti‐p‐AKT (ab192623, 1/1000), anti‐mTOR (ab134903, 1/10000), anti‐p‐mTOR (ab137133, 1/10000), and anti‐GAPDH (ab181602, 1/10000) antibodies overnight at 4°C, followed by goat anti‐rabbit IgG culture (ab96899, 1/10000) at room temperature for 4 h. We analyzed protein levels by using an XR gel imaging analysis system (Bio‐Rad, USA) after visualizing the ECL kit (Thermo Scientific, China). All antibodies were purchased from Abcam.

### 2.7 Enzyme‐linked immunosorbent assay

The colon or hippocampus tissue was homogenized with pre‐cooled PBS, respectively, and then the supernatant was obtained from homogenate centrifugated with 5000 × g for 5–10 min at 4 °C. Also, the serum samples were obtained from caudal venous blood centrifugated with 5000 × g for 5–10 min at 4 °C. ELISA was used to determine the levels of cytokines, namely TNF‐α, IL‐1β, and IL‐6, in the colon, serum, and hippocampus. TNF‐α (ab108913), IL‐1β (ab255730), and IL‐6 (ab234570) kits were purchased from Abcam (UK). The unit of concentration was recorded in a picogram per milliliter.

### 2.8 Fecal sample collection

Fresh fecal samples were collected from rats after they were starved for 12 h, and the collected samples were transferred to Eppendorf tubes supplemented with a drop of sodium azide (1:100, v/v) per tube, followed by flash‐freezing in liquid nitrogen for 15 min. The frozen samples were stored at −80°C for further tests.

### 2.9 16S rDNA sequencing

Fecal samples of the rats were collected, and the total bacterial DNA of gut microbiota was extracted through Fast DNA SPIN kit (MP Biomedicals, USA) after starving the rats for 12 h. Bacterial genomic DNA was assessed using Oebiotech (Shanghai, China). The V3‐V4 region of 16S rDNA was amplified using PCR with the following primers: 343F (5’‐TAC GGR AGG CAG CAG‐3′) and 798R (5′‐AGG GTA TCT AAT CCT‐3′) and then sequenced through Illumina MiSeq platform (Illumina, USA) according to the manufacturer's instructions.

### 2.10 Bioinformatic analysis for gut microbiota

We investigated the community alteration of gut microbiota among the three groups based on operational taxonomic units (OTUs), community structure, α‐diversity, and β‐diversity. Indices of α‐diversity included Chao1, Shannon, and Simpson. We discuss β‐diversity according to the simple distance obtained from the Bray–Curtis method and PCoA. We used linear discriminant analysis coupled with effect size measurements (LEfSe) to assess the altered microbiota among the groups. We predicted the functional categories by using the phylogenetic investigation of communities through the reconstruction of unobserved states (PICRUSt). Additionally, Spearman's analysis demonstrated the relationship between metabolites/microbiota and depression‐like behaviors/inflammation/pathways.

### 2.11 GC–MS

GC–MS analysis was performed using Oebiotech (Shanghai, China). Prior to GC–MS analysis, the fecal samples stored at −80°C were transferred to Eppendorf tubes containing 500 μl of water and centrifuged at 10,000 × g for 15 min. We used the Agilent 7890B gas chromatography system coupled with the Agilent 5977A MSD system (Agilent, USA) to determine metabolites in gut microbiota. The DB‐5MS‐fused silica capillary column (30 m × 0.25 mm × 0.25 μm, Agilent, USA) was used to separate the samples. Helium (>99.999%) was used as the carrier gas at a constant flow rate of 1 ml/min through the column. The oven temperature for GC–MS was initially set to 60°C for 30 s, which was ramped to 125°C at a rate of 8°C per min, 210 °C at a rate of 5°C per min, 270°C at a rate of 10°C per min, 305°C at a rate of 20°C per min, and finally held at 305°C for 5 min. The temperature of MS quadrupole and ion source (electron impact) was set to 150°C and 230°C, respectively. The collision energy was 70 eV. Mass spectrometric data were acquired in a full‐scan mode from 50 to 500 m/z.

### 2.12 Metabolome analysis

Orthogonal partial least squares discriminant analysis (OPLS‐DA) was used to distinguish the altered metabolites among the groups. We determined the altered metabolites according to the variable importance of projection (VIP) > 1 and *p* value <0.05 and visualized the result by using volcano plots based on ggplot2. The fold change value of >1 indicated upregulation, and that of <1 indicated downregulation.

### 2.13 Statistical analysis

Experiment data are expressed as mean ± SEM, followed by visualization using GraphPad Prism 8.0 and using SPSS 22.0 (IBM, USA). Experiment data were subject to test for normality by Shapiro–Wilk test and then statistical analysis was performed with one‐way analysis of variance, followed by Dunnett's multiple comparison test. *p* value <0.05 with a 95% confidence interval indicated a significant difference among the groups.

## 3 RESULTS

### 3.1 Effect of SGKL on depression‐like behaviors of CRS‐stimulated rats

**FIGURE 1:**
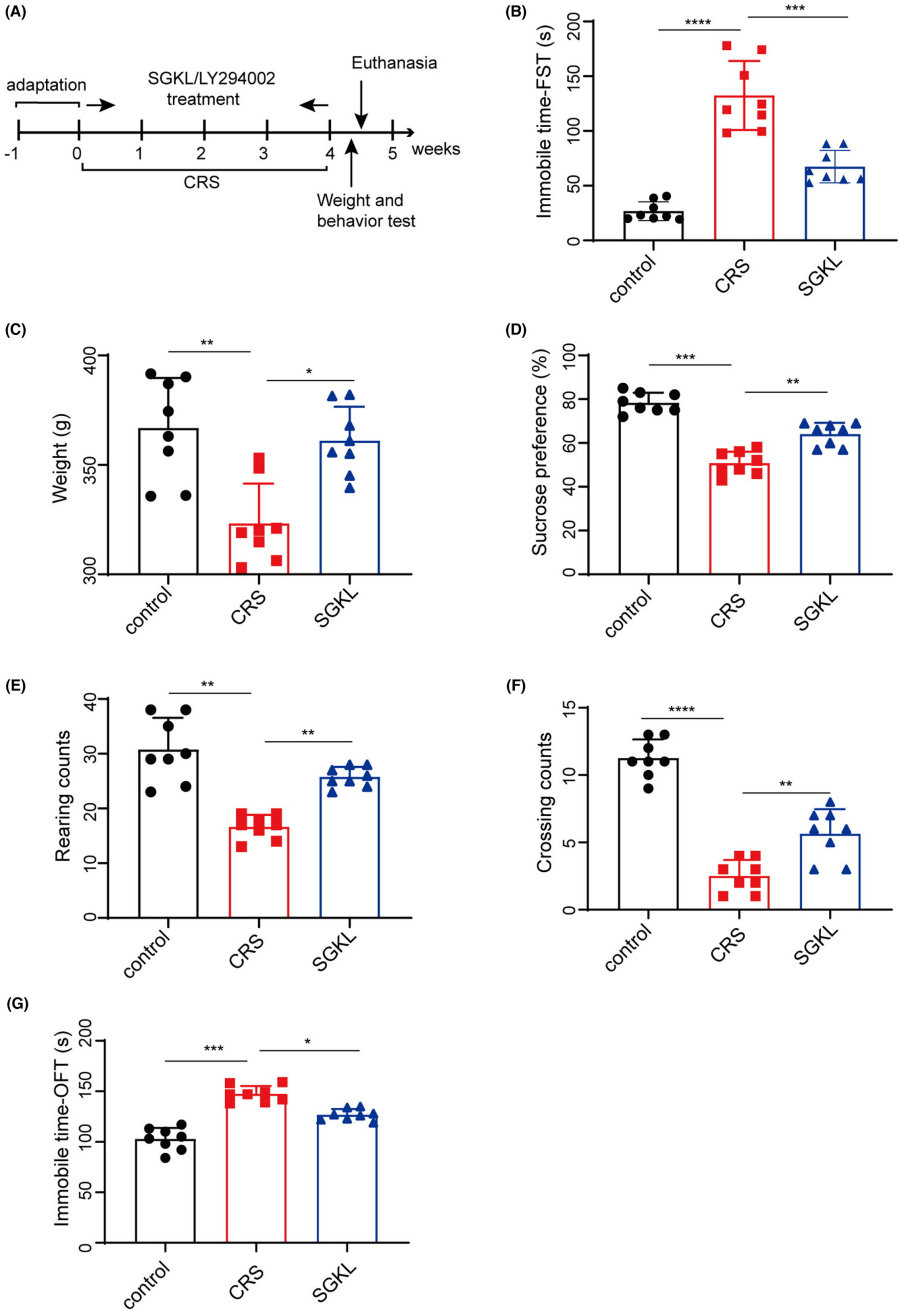
Effect of SGKL on behaviors of CRS‐evoked mice. After the adaptation to a new environment for 1 week, rats (*n* = 8) were stimulated with CRS to evoke depression‐like behaviors for 4 weeks, during which these were rats treated with SGKL. (A) Experimental timeline of CRS stimulation to rats. (B) immobile time of forced swimming, Control versus CRS (*p* < 0.0001, 95% CI: 81.18 to 130.2), CRS versus SGKL (*p* < 0.001, 95% CI: 40.47 to 89.53). (C) body weight, Control versus CRS, (*p* < 0.01, 95% CI: −66.24 to −20.92), CRS versus SGKL (*p* < 0.05, 95% CI: −60.52 to −15.20). (D) sugar preference, Control versus CRS (*p* < 0.001, 21.27 to 33.98), CRS versus SGKL (*p* < 0.01, 95% CI: −19.60 to −6.899). (E) rearing counts of the open‐field experiment, Control versus CRS (*p* < 0.01, 95% CI: −18.55 to −9.699), CRS versus SGKL (*p* < 0.01, 95% CI: −13.55 to −4.699). (F) crossing counts of the open‐field experiment, Control versus CRS (*p* < 0.0001, 95% CI: −10.53 to −6.970), CRS versus SGKL (*p* < 0.01, 95% CI: −4.905 to −1.345). (G) immobile time in the corner of the open‐field experiment, Control versus CRS (*p* < 0.001, 95% CI: 34.57 to 54.68), CRS versus SGKL (*p* < 0.05, 95% CI: 10.57 to 30.68). *p < 0.05, **p < 0.01, ***p < 0.001, ****p < 0.0001

After the rats were adapted to a new environment for 1 week, they were stimulated with CRS to evoke depression‐like behaviors for 4 weeks, during which they were treated with SGKL. Their body weight and depression‐like behavior were recorded weekly after modeling (Figure 1A). CRS evoked the increase in immobile time of FST compared with that of the control group, whereas treatments with SGKL significantly repressed the increased immobile time (Figure 1B, F = 53.09, *p* < 0.0001). Significant elevations in the body weight (Figure 1C, F = 12.28, *p* = 0.0003), sugar preference (Figure 1D, F = 60.13, *p* < 0.0001), rearing counts (Figure 1E, F = 29.43, *p* < 0.0001), and crossing counts (Figure 1F, F = 69.72, *p* < 0.0001) were observed after CRS stimulation in the rats. However, treatments with SGKL suppressed these parameters in the CRS‐stimulated rats. Furthermore, SGKL treatment reduced the CRS‐elevated immobile time of OFT (Figure 1G, F = 55.39, *p* < 0.0001). Collectively, we determined that SGKL could significantly improve depression‐like behaviors of CRS‐stimulated rats.

### 3.2 Effect of SGKL on inflammation in CRS‐stimulated rats through microglial inactivation

**FIGURE 2:**
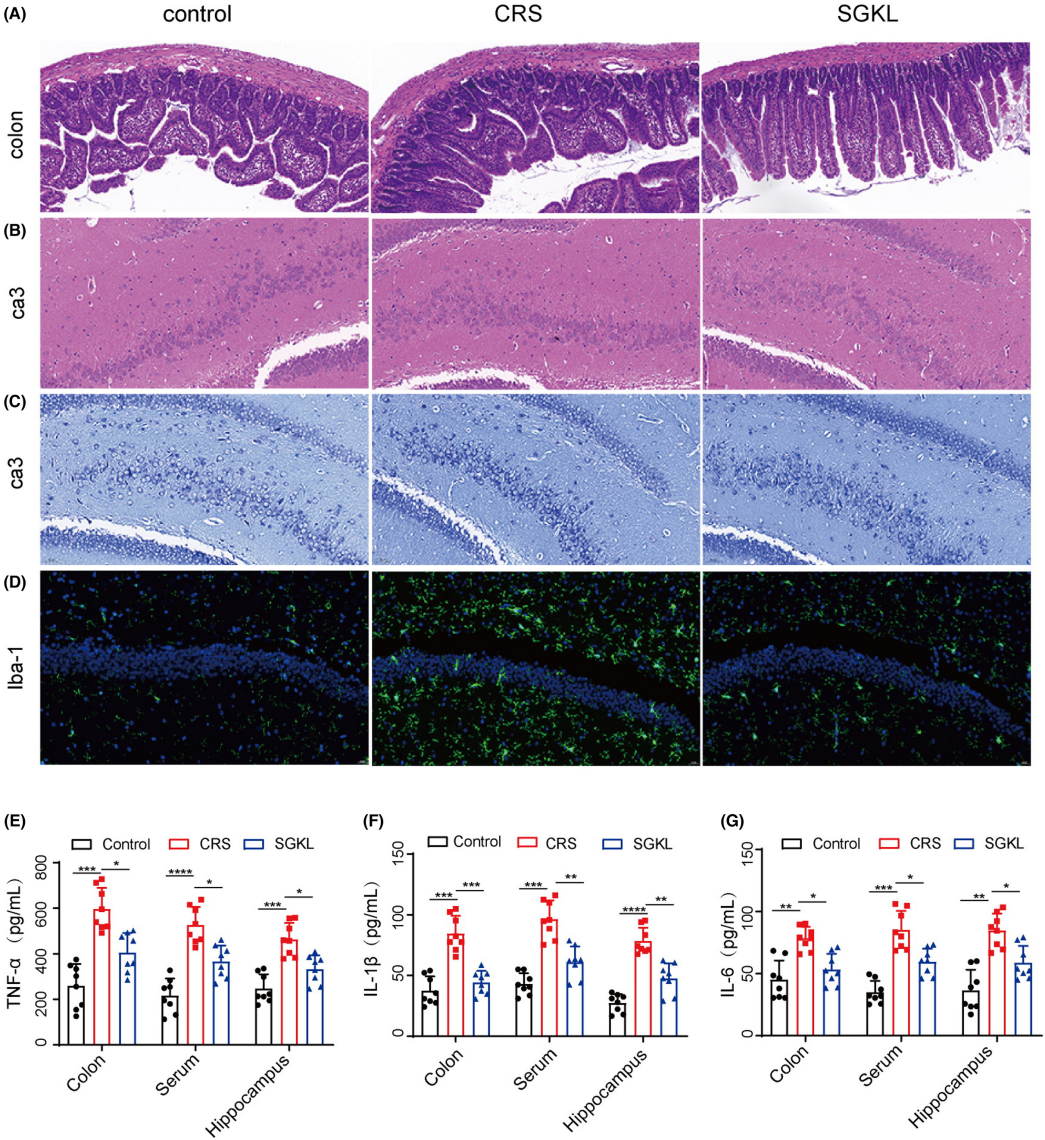
Effect of SGKL on inflammation in CRS‐stimulated rats through microglial inactivation. (A) Histopathological changes in the colon determined through HE staining (scale = 50 μm). (B) Histopathological changes in hippocampus CA3 determined through HE staining (scale = 50 μm). (C) Alterations of neuron in the hippocampus CA1. (D) Altered activities of microglia determined through immunofluorescence (scale = 50 μm). (E) The levels of TNF‐α in the colon, serum, and hippocampus measured through ELISA. For colon, Control versus CRS (*p* < 0.001, 95% CI: −447.4 to −226.4), CRS versus SGKL (*p* < 0.05, 95% CI: 79.88 to 300.8). For serum, Control versus CRS (*p* < 0.0001, 95% CI: −400.0 to −218.2), CRS versus SGKL (*p* < 0.05, 95% CI: 69.76 to 251.8). For the hippocampus, Control versus CRS (*p* < 0.001, 95% CI: −295.2 to −137.0), CRS versus SGKL (*p* < 0.05, 95% CI: 52.05 to 210.3). F. The levels of IL‐1β in the colon, serum, and hippocampus measured through ELISA. For colon, Control versus CRS (*p* < 0.001, 95% CI: −66.72 to −27.43), CRS versus SGKL (*p* < 0.001, 95% CI: 20.65 to 59.95). For serum, Control versus CRS (*p* < 0.001, 95% CI: −74.78 to −31.97), CRS versus SGKL (*p* < 0.01, 95% CI: 14.00 to 56.82). For the hippocampus, Control vs CRS (*p* < 0.0001, 95% CI: −68.84 to −33.46), CRS versus SGKL (*p* < 0.01, 95% CI: 13.06 to 48.44). G. The levels of IL‐6 in the colon, serum, and hippocampus measured through ELISA. For colon, Control versus CRS (*p* < 0.01, 95% CI: −54.01 to −12.92), CRS versus SGKL (*p* < 0.05, 95% CI: 4.53 to 45.62). For serum, Control versus CRS (*p* < 0.001, 95% CI: −69.56 to −30.70), CRS versus SGKL (*p* < 0.05, 95% CI: 6.12 to 44.99). For the hippocampus, Control versus CRS (*p* < 0.01, 95% CI: −72.04 to −24.48), CRS vs. SGKL (*p* < 0.05, 95% CI: 2.18 to 49.74). *p < 0.05, **p < 0.01, ***p < 0.001, ****p < 0.0001

We investigated the role of SGKL in histopathological alterations in the colon and hippocampus CA3 area through HE staining. IBA‐1 immunofluorescence was used to determine microglial cell activation and Franna Nissl assay to assess neuron alteration. Levels of inflammatory cytokines, TNF‐α, IL‐1β, and IL‐6, in the colon, serum, and hippocampus were measured through ELISA. Compared with the control group, the colon tissue in the CRS group displayed a disordered arrangement of colon gland cells and intestinal mucosa with impaired integrity (Figure 2A). CRS‐stimulated rats exhibited triangular or polygonal cells, shrinkage in the nuclear membrane, and unclear nucleolus in the hippocampus CA3 area compared with the control group rats (Figure 2B). Compared with the CRS group, rats in the SGKL group had intestinal mucosa with better integrity and well‐arranged cells in the colon tissue and hippocampus CA3 area (Figure 2A & 2B). As shown in Figure 2C and D, CRS evoked the activation of microglial cells and the decreased survival of neurons in the rats. Nonetheless, the treatment with SGKL resumed the survival of neuron cells in CRS‐stimulated rats and induced the inactivation of microglial cells. Importantly, treatments with SGKL repressed the increased levels of TNF‐α, IL‐1β, and IL‐6 in the colon, serum, and hippocampus in response to CRS stimulation in rats (Figure 2E, F_1_ = 27.12, *p* < 0.0001, F_2_ = 33.56, *p* < 0.0001, F_3_ = 21.97, *p* < 0.0001; Figure 2F, F_1_ = 34.99, *p* < 0.0001, F_2_ = 36.66, *p* < 0.0001, F_3_ = 48.26, *p* < 0.0001; Figure 2G, F_1_ = 14.98, *p* < 0.0001, F_2_ = 34.70, *p* < 0.0001, F_3_ = 21.52, *p* < 0.0001). Therefore, we demonstrated that SGKL evokes the inactivation of microglial cells to repress the inflammation‐induced neuron damage in CRS‐stimulated rats.

### 3.3 Effect of SGKL on gut microbiota alteration in CRS‐stimulated rats

**FIGURE 3:**
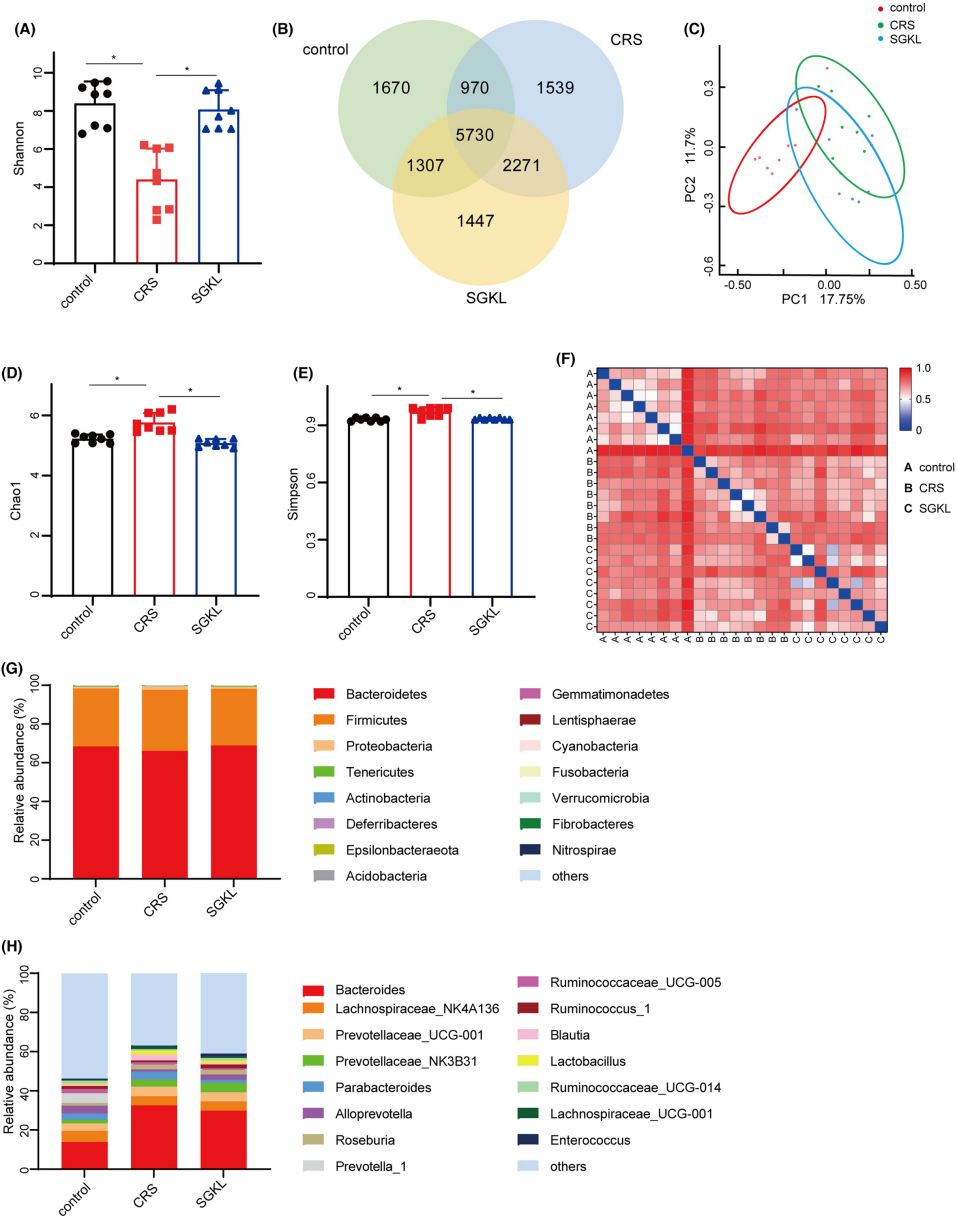
Effect of SGKL on gut microbiota alteration in CRS‐evoked rats. Fecal samples from rats were collected for 16S RNA analysis. (A) Shannon, Control versus CRS (*p* = 0.0177, 95% CI: −7.08 to −0.89), CRS versus SGKL (*p* = 0.0255, 95% CI: −6.76 to −0.57). (B) OTUs. (C) PCoA. (D) Chao1 (*p* = 0.0323, 95% CI: 0.06–1.02). (E) Simpson (*p* = 0.012, 95% CI: 0.21–1.17). (F) Bray–Curtis distance. (G) Relative contribution of fecal microbiota at the phylum level. (H) Relative contribution of fecal microbiota at the genus level

**FIGURE 4:**
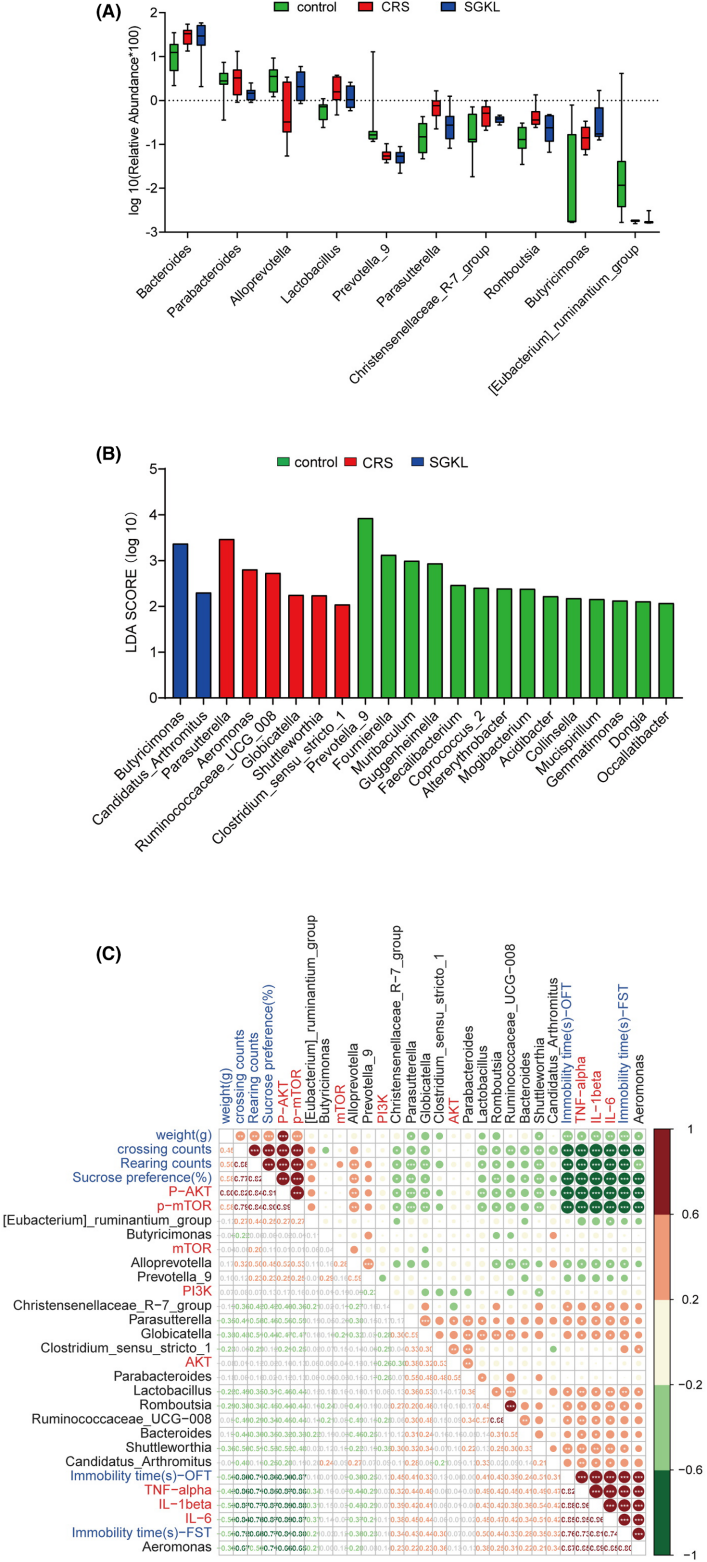
The correlation between gut microbiota and behaviors, inflammation in the hippocampus or PI3K/Akt/mTOR pathway. (A) The top 10 phyla sorted by the relative abundance of the altered microbiota among control, CRS, and SGKL groups based on the Kruskal–Wallis method. (B) Linear discriminant analysis effect size (LEfSe) analysis on genus level among the control, CRS, and SGKL groups. (C) Spearman analysis for the correlation between gut microbiota and behaviors, inflammation in the hippocampus, or PI3K/Akt/mTOR pathway

16S RNA sequencing was used to investigate the effect of SGKL in gut microbiota alteration in CRS‐stimulated rats. Treatment with SGKL elevated the CRS‐decreased α‐diversity based on Shannon (Figure 3A, F = 23.77, *p* < 0.0001), Chao1 (Figure 3D, F = 23.72, *p* < 0.0001), and Simpson (Figure 3E, F = 17.13, *p* < 0.0001) and shifted CRS‐induced β‐diversity through PCoA (Figure 3C) based on the Bray–Curtis distance (Figure 3F). A significant alteration in OTUs among the control group, CRS group, and SGKL group was observed (Figure 3B). At the phylum level, two major phyla in gut microbiota were *Bacteroidetes and Firmicutes*. Among the three groups, there was no difference in the two phyla (Figure 3G). We calculated the ratio of firmicutes/Bacteroidetes at the phylum level. The ratio of firmicutes/Bacteroidetes in the control group, CRS group, and SGKL group were 0.45 ± 0.12, 0.50 ± 0.20, and 0.51 ± 0.39. Among the three groups, there is no statistical difference in the ratio of firmicutes/Bacteroidetes (*p* = 0.8869, F = 0.1208). The five major genera in gut microbiota at the genus level were Bacteroides, Lachnospiraceae_NK4A136, Prevotellaceae_UGG‐001 *Prevotellaceae*_NK3B31, and *Parabacteroides*. We analyzed the altered microbiota among the three groups based on the Kruskal–Wallis method. The top 10 genera sorted by relative abundance in the altered microbiota were as follows: Bacteroides, Parabacteroides, Alloprevotella, Lactobacillus, Prevotella_9 *Parasutterella*, *Christensenellaceae_R‐7_group*, *Romboutsia*, *Butyricimonas,* and *[Eubacterium]_ruminantium_group* (Figure 4A). According to linear discriminant analysis effect size (LEfSe), *Butyricimonas* and *Candidatus Arthromitus* were enriched in the SGKL‐treated rats, whereas six genera, namely *Parasutterella*, *Aeromonas*, *Ruminococcaceae_UCG_008*, *Globicatella*, *Shuttleworthia,* and *Clostridium_sensu_stricto_1*, were enriched in CRS‐stimulated rats (Figure 4B). Based on PICRUSt, gut microbiota might be majorly involved in five functional categories: general function prediction, carbohydrate transport and metabolism, transcription, amino acid transport and metabolism, and cell wall/membrane/envelope biogenesis (Figure S2).

We investigated Spearman correlations among altered microbiotas, behaviors, inflammation, and PI3K/Akt/mTOR pathway (Figure 4C). We analyzed differences among 16 altered genera. For example, a positive correlation between *Clostridium_sensu_stricto_1* and PI3K/Akt/mTOR pathway was observed. *Parabacteroides* was positively associated with weight; however, it was negatively associated with the PI3K/Akt/mTOR pathway. *[Eubacterium]_ruminantium_group* was the positive factor of rearing counts and the negative one of inflammation in the hippocampus. We observed positive correlations of *Globicatella* with FST immobility time, sucrose preference, and inflammation in the hippocampus, and it negatively affected the rearing counts, crossing counts, and PI3K/Akt/mTOR pathway. *Alloprevotella* was positively correlated with the rearing counts and PI3K/Akt/mTOR pathway and negatively associated with the immobility time of OFT/FST, sucrose preference, and inflammation in the hippocampus. *Ruminococcaceae_UCG‐008* exhibited negative relations with crossing counts and PI3K/Akt/mTOR pathway and a positive association with immobility time of OFT and inflammation in the hippocampus. *Parasutterella* and *Romboutsia* were negatively related to rearing counts, crossing counts, and PI3K/Akt/mTOR pathway. These two genera were positively associated with immobility time of OFT/FST, sucrose preference, and inflammation in the hippocampus. *Aeromonas* and *Shuttleworthia* were positively associated with immobility time of OFT/FST, sucrose preference, and inflammation in the hippocampus but negatively associated with the weight, rearing counts, crossing counts, and PI3K/Akt/mTOR pathway. *Lactobacillus* was positively related to the immobility time of FST. *Christensenellaceae_R‐7_group* was positively correlated with immobility time of sucrose preference and negatively associated with the rearing counts, crossing counts, and PI3K/Akt/mTOR pathway; however, they were positively correlated with immobility time of OFT and inflammation in the hippocampus. *Candidatus_Arthromitus* and *Bacteroides* negatively affected the crossing counts and were positively associated with inflammation in the hippocampus. Bacteroides was positively correlated with sucrose preference. Collectively, altered microbiota was associated with the behavior, inflammation in the hippocampus, and PI3K/Akt/mTOR pathway.

### 3.4 Effect of SGKL in metabolites of CRS‐stimulated rats

**FIGURE 5:**
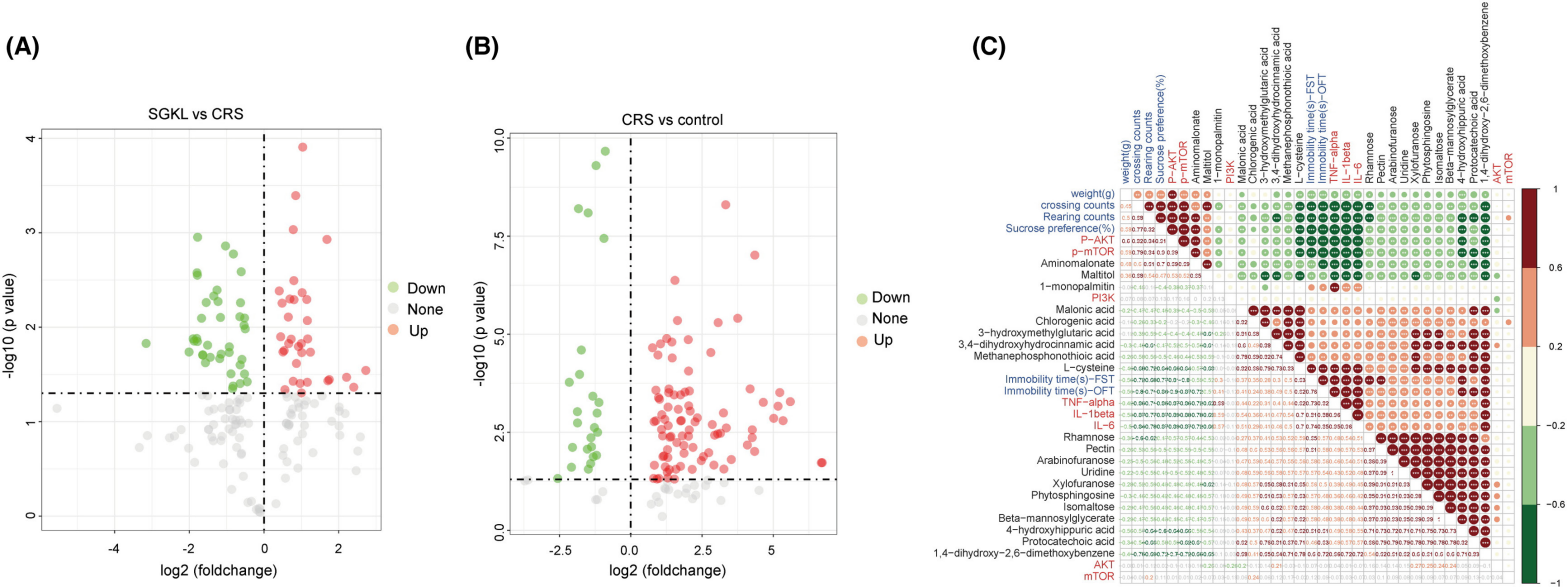
Effect of SGKL on altered metabolites in CRS‐stimulated rats. Fecal samples from rats were collected for GC–MS analysis. (A) The altered metabolites in the SGKL group compared with those in the CRS group. (B) Altered metabolites between the CRS group and the control group. (C) Spearman correlation for the relation of altered metabolites with behaviors, inflammation in the hippocampus, and PI3K/Akt/mTOR pathway

Interestingly, 34 of the altered metabolites were upregulated, whereas 40 of the altered metabolites were downregulated in the SGKL group compared with those in the CRS‐stimulated rats (Figure 5A and Table S1). The CRS group had 114 of the altered metabolites (89 upregulated and 25 downregulated) compared with the control group (Figure 5B and Table S2). Taking the intersection of altered metabolites, the top 20 of 30 metabolites were included in the Spearman correlation analysis to determine the relationship of altered metabolites with behaviors, inflammation in the hippocampus, and the PI3K/Akt/mTOR pathway (Figure 5C). For instance, weight was positively correlated with aminomalonate and maltitol, whereas it was negatively correlated with L‐cysteine, rhamnose, 4‐hydroxyhippuric acid, and 1,4‐dihydroxy‐2,6‐dimethoxybenzene. In addition, aminomalonate and maltitol were negatively associated with the hippocampus inflammation factors and immobility times of OFT/FST. Counts of OFT (rearing and crossing), sucrose preference, and the PI3K/Akt/mTOR pathway were positively associated with aminomalonate and maltitol but negatively related to other metabolites, including chlorogenic acid. Inflammation in the hippocampus was positively affected by metabolites, in addition to aminomalonate and maltitol. Generally, differential metabolites were associated with depression‐like behaviors, inflammation, and PI3K/Akt/mTOR pathway. Collectively, we demonstrated that SGKL improved depression‐like behaviors and inflammation in CRS‐stimulated rats through different metabolites.

### 3.5 Effect of SGKL in depression through the PI3K/Akt/mTOR pathway in vitro/vivo

**FIGURE 6:**
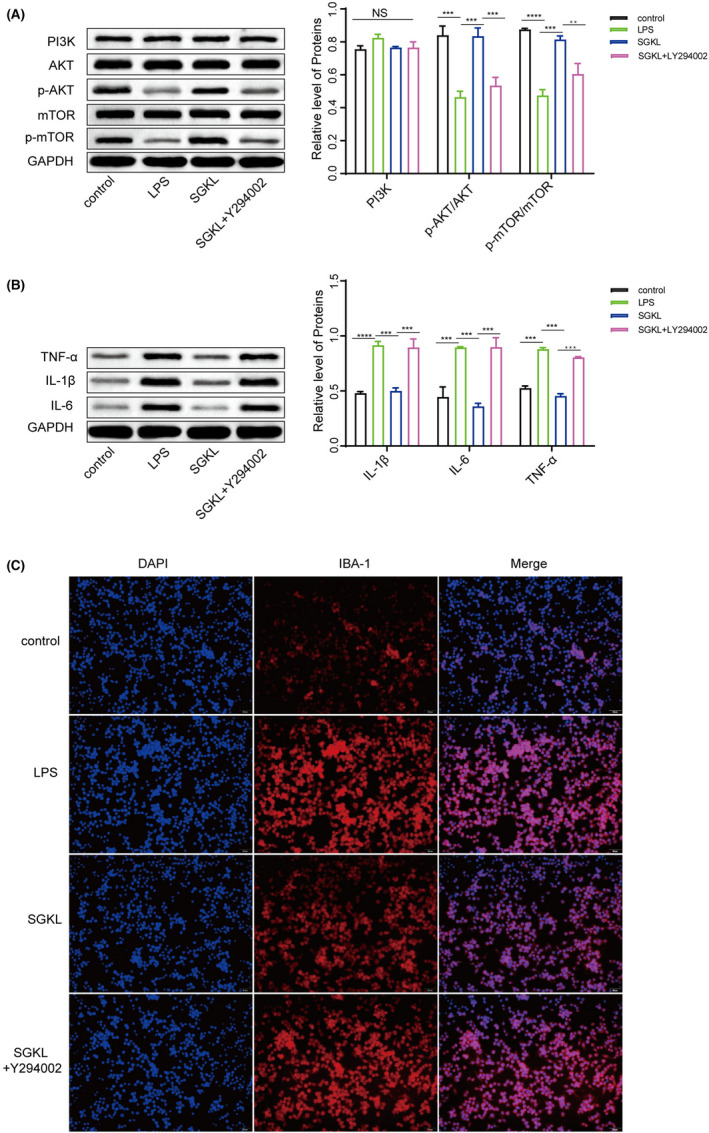
Effect of SGKL on LPS‐stimulated microglia through the PI3K/Akt/mTOR pathway in vitro. Microglial cells were stimulated with LPS and then treated with SGKL, in which LY294002 inhibited the activity of PI3K. (A) Protein levels of PI3K, Akt, p‐Akt, mTOR, and p‐mTOR determined through western blotting. (B) Protein levels of TNF‐α, IL‐1β, and IL‐6 determined through western blotting. (C) Activities of microglia determined through immunofluorescence (scale = 50 μm). NS, *p* > 0.05. *p < 0.05, **p < 0.01, ***p < 0.001, ****p < 0.0001

The effects of SGKL in LPS‐stimulated microglial cells mediated through the PI3K/Akt/mTOR pathway were examined. Microglial cells were stimulated with LPS and then treated with an SGKL dose of 7.11 mg/mL for 72 h, in which LY294002 inhibited the activity of PI3K. Elevated phosphorylation of the PI3K/Akt/mTOR pathway was observed in LPS‐stimulated microglia with SGKL treatment, and LY294002 evoked the inactivation of the PI3K/Akt/mTOR pathway to reverse the SGKL‐induced effect in LPS‐stimulated microglial cells (Figure 6A), suggesting that SGKL produced the activation of the PI3K/Akt/mTOR pathway in LPS‐stimulated microglial cells. SGKL‐induced reductions in the TNF‐α, IL‐1β, and IL‐6 levels were suppressed by LY294002 in LPS‐stimulated cells (Figure 6B), implying that SGKL improved inflammation in LPS‐stimulated microglia through PI3K/Akt/mTOR pathway activation. SGKL treatment inhibited the LPS‐increased activation of microglia; LPS‐stimulated microglial cells with SGKL treatment were activated by LY294002 (Figure 6C), indicating SGKL repressed the activation of LPS‐evoked microglial cell through the PI3K/Akt/mTOR pathway.

**FIGURE 7:**
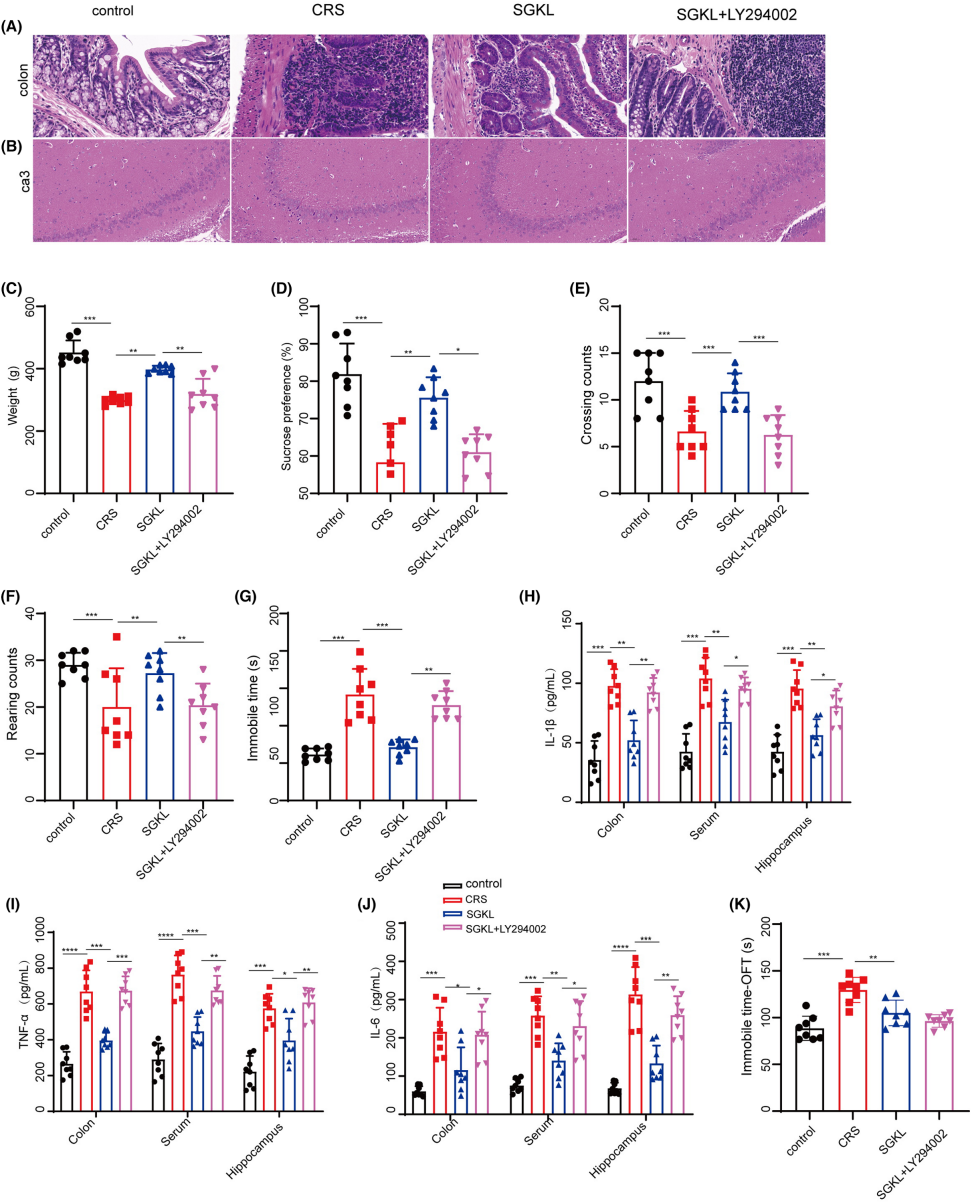
Effect of SGKL on depression rats evoked by CRS through the PI3K/Akt/mTOR pathway. Rats (*n* = 8) were stimulated with CRS to evoke depression‐like behaviors for 4 weeks, during which they were treated with SGKL. LY294002 is the inhibitor of PI3K. (A) Histopathological changes in the colon determined through HE staining (200X, scale = 50 μm). (B) Histopathological changes in the hippocampus CA3 determined through HE staining (200X, scale = 50 μm). (C) Body weight, Control versus CRS (*p* < 0.001, 95%CI: 94.79 to 211.6), CRS versus SGKL (*p* < 0.01, 95% CI: −156.8 to −39.99), SGKL versus SGKL+LY294002 (*p* = 0.0071 95% CI:19.99 to 136.8). (D) Sugar preference, Control versus CRS (*p* = 0.0007, 95% CI: 9.99–37.00), CRS versus SGKL (*p* = 0.0094, 95% CI: −30.98 to −3.975), SGKL versus SGKL+LY294002 (*p* = 0.0298, 95% CI: 1.255 to 28.26). (E) Crossing counts of the open‐field experiment, Control versus CRS (*p* < 0.001, 95% CI: 5.19 to 13.61), CRS versus SGKL (*p* = 0.0019, 95% CI: −10.18 to −2.39), SGKL versus SGKL+LY294002 (*p* = 0.0044, 95% CI: 1.79 to 10.21). F. Rearing counts of the open‐field experiment, Control versus CRS (*p* = 0.0005, 95% CI: 33.41 to 58.99), CRS versus SGKL (*p* < 0.001 95% CI: −52.99 to −27.41), SGKL versus SGKL+LY294002 (*p* < 0.001, 95% CI: 18.81 to 44.39). (G) Immobile time of forced swimming, Control versus CRS (*p* < 0.001, 95% CI: −113.3 to 47.00), CRS versus SGKL (*p* < 0.001, 95% CI: 37.25 to 103.5), SGKL versus SGKL+LY294002 (*p* < 0.01, 95% CI: −89.39 to −23.12). (H–J). The levels of TNF‐α, IL‐1β, and IL‐6 in the colon, serum, and hippocampus determined through ELISA. (K) Immobile time in the coner of open field experiment, Control versus CRS (*p* < 0.001, 95% CI: ‐70.26 to 39.58), CRS versus SGKL (*p* < 0.001, 95% CI: 24.80 to 55.48), SGKL versus SGKL+LY294002 (*p* = 0.4367, 95% CI: ‐6.77 to 23.91). *p < 0.05, **p < 0.01, ***p < 0.001, ****p < 0.0001

We investigated the role of SGKL in the PI3K/Akt/mTOR pathway. SGKL‐improved histological alterations in the colon tissue and hippocampus CA3 of CRS rats were significantly reversed by LY294002 (Figure 7A&7B). LY294002 repressed SGKL‐evoked elevations of body weight, sugar preference, rearing/crossing counts, and immobile time of OFT in the open‐field experiment (Figure 7C, F = 38.78, *p* < 0.0001; Figure 7D, F = 18.29, *p* < 0.0001; Figure 7E, F = 12.28, *p* < 0.0001; Figure 7F, F = 5.98, *p* = 0.0028; Figure 7K, F = 38.70, *p* < 0.0001). Also, LY294002 increased the SGKL‐reduced immobile time of the forced swimming test (Figure 7G, F = 30.43, *p* < 0.0001) in the CRS‐stimulated rats. For inflammation in the CRS‐stimulated rats, LY294002 increased SGKL‐inhibited levels of TNF‐α, IL‐1β, and IL‐6 (Figure 7H, F_1_ = 33.62, *p* < 0.0001, F_2_ = 25.74, *p* < 0.0001, F_3_ = 22.61, *p* < 0.0001; Figure 7I, F_1_ = 49.60, *p* < 0.0001, F_2_ = 46.06, *p* < 0.0001, F_3_ = 28.81, *p* < 0.0001; Figure 7J, F_1_ = 15.93, *p* < 0.0001, F_2_ = 24.64, *p* < 0.0001, F_3_ = 40.52, *p* < 0.0001;), suggesting that the PI3K/Akt/mTOR pathway mediated SGKL‐regulated inflammation in CRS‐stimulated rats. Collectively, the results indicated that the PI3K/Akt/mTOR pathway is the target of SGKL treatment that mediates the improvement of depression‐like behavior and inflammation in CRS rats.

## 4 DISCUSSION

MDD is an inhomogeneous illness with a complex and multifactorial etiopathogenesis involved in psychological, physiological, genetic, and social environments, leading to a lack of effective management for patients with MDD.^36^, ^37^ Metabolomics based on the analysis of small molecular metabolites is involved in the pathological network of MDD that links metabolic phenotypes to MDD symptoms.^38^ In this investigation, we first demonstrated based on a comprehensive analysis that SGKL treatment significantly contributes to the improvement of depression‐like phenotypes in CRS‐stimulated rats by altering metabolites associated with gut microbiota, in which the PI3K/Akt/mTOR pathway is the key therapeutic target of SGKL treatment that mediates metabolite changes, inflammation, and microglial activation.

First, SGKL treatment decreased the CRS‐increased immobile time and elevated the CRS‐repressed alterations of body weight, sugar preference, and rearing/crossing counts in the open‐field experiment, which revealed a significant role of SGKL treatment in depression‐like behaviors. Significant alleviations of hippocampus damage and neuron impairment due to SGKL treatment in CRS rats were observed, which indicated that SGKL treatment may be involved in depression‐dependent neurobiology. Microglia and neuroinflammation play critical roles in the development of depression symptoms.^39^ We discovered that SGKL treatment significantly inhibited the CRS‐induced activation of microglia and CRS‐elevated neuroinflammation. Microglial‐associated inflammation provides a conducive environment for MDD development. Elevated inflammation induces the activation of microglia to trigger subsequent dysregulation of biological pathways that links to neuron dysfunction in depression.^40^ A previous report^41^ revealed an increased activation of microglia with elevated levels of TNF‐α and IL‐6 in MDD. Thus, the SGKL treatment‐induced reduction in microglial‐associated neuroinflammation explains the mechanism of hippocampus lesion in CRS‐stimulated rats at the cellular level. In particular, SGKL treatment in CRS‐stimulated rats was found to improve the impaired intestinal barrier, according to the analysis of colonic histopathology and inflammation. We discovered a dynamic interaction between the intestinal barrier and gut microbiota.^42^ Relatively, an impaired intestinal barrier in depression indicates a significant alteration of gut microbiota. Gut microbiota and metabolites are infiltrated into blood circulation through the impaired intestinal barrier, leading to cytokine activation and systemic inflammation that are crucial to behaviors. Our findings indicate the potential of SGKL treatment in gut microbiota associated with depressive phenotypes.

Then, we analyzed fecal microbiota diversity in feces based on 16S RNA‐sequencing analysis to explore the role of SGKL in CRS‐stimulated gut microbiota. SGKL treatment shifted CRS‐induced microbiota competition based on OTUs, Shannon index, Chao1 index, and Simpson index. The altered populations of gut microbiotas according to LEfSe may be related to the development of depressive phenotypes during SGKL treatment. The enrichment of *Parasutterella*, *Aeromonas*, *Ruminococcaceae_UCG_008*, *Globicatella*, *Shuttleworthia*, and *Clostridium_sensu_stricto_1* in CRS rats indicates that these microbiotas may be the MDD biomarkers. Particularly, most of these genera were significantly associated with behaviors and inflammation in the hippocampus. *Candidatus Arthromitus* in SGKL‐treated rats may be the therapeutic target of SGKL for improving behaviors because of the positive effect of SGKL on inflammation and the negative effect on behavior. *Candidatus Arthromitus* in the gut is involved in the CRS‐induced phenotype, revealing the potential role in regulating the progression of CRS‐induced depression in the *Candidatus Arthromitus*‐associated gut microbiota environment.^43^ It may also be associated with the occurrence and development of depression through 3‐methyldioxyindole.^44^ In other words, *Candidatus Arthromitus* monitors the altered metabolites in MDD, which develop various metabolic lesion phenotypes and subsequently evoke mood disorder or physiological dysfunction. This may be the major reason for the improvement in depressive phenotype MDD with SGKL treatment through alteration of gut microbiota. It remains unclear how *Candidatus Arthromitus* is related to depression‐involved metabolites, which warrants further investigations based on fecal microbiota transplantation.

A mechanism that links microbiota/metabolites in the gut to microglia might exist. PI3K/Akt/mTOR pathway is the target of SGKL based on the network pharmacology (data not shown), providing a hypothesis that it is the molecular approach linking SGKL treatment to metabolite variation in MDD. Meanwhile, we discovered that the PI3K/Akt/mTOR pathway was significantly associated with altered gut microbiota, metabolites, behaviors, and inflammation in the hippocampus. These findings suggest that, during SGKL treatment, the PI3K/Akt/mTOR pathway may be involved in mediating the pathological mechanism of altered gut microbiota for the depression process. In the cellular process, mTOR is activated by PI3K/Akt pathway to play a regulatory role in survival, growth, proliferation, and metabolism.^45^ Interestingly, SGKL treatment seemly targets PI3K/Akt/mTOR pathway to regulate microglial activation during LPS‐induced inflammatory microglia. Possibly, SGKL improves behaviors in CRS‐induced rats targeting PI3k/Akt/mTOR pathway via two pathways: a) SGKL alters the composition structure of gut microbiota to target the PI3K/Akt/mTOR pathway in the brain, thereby regulating depression progression; b) transported by the circulatory system, SGKL may be delivered to microglia and then target PI3K/Akt/mTOR pathway‐mediated microglial activation and inflammation. Roughly, SGKL‐regulated PI3K/Akt/mTOR pathway may play a critical role in the gut–brain axis.

How dose PI3K/Akt/mTOR pathway effect on depression progression via activated microglia? There are two potential mechanisms. On the one hand, the PI3K/Akt/mTOR pathway playing an antidepressant role is dependent on the communication between mTOR and N‐methyl‐D‐aspartate receptor (NMDAR), which means that the practical part of antidepressants is based on the activation of mTOR.^46^, ^47^ On the other hand, the PI3K/Akt/mTOR pathway regulates the metabolic phenotype of microglia and glia‐mediated neuroinflammation via altering energy metabolism remodeling^48^ and mediating the production of nitric oxide,^49^, ^50^ thereby triggering microglial‐associated depression pathogenesis. We inferred that SGKL treatment might activate the PI3K/Akt/mTOR pathway that mediates the regulation of gut microbiota alteration toward microglia to evoke metabolic reprogramming and NMDAR‐associated neurobiology MDD at the molecular level, which is required for a follow‐up work based on fecal microbiota transplantation. According to Spearman correlation, the correlation between PI3K/Akt/mTOR pathway and gut microbiota or metabolites can explain how SGKL treatment affects the PI3K/Akt/mTOR pathway activity in the hippocampus during depression progression. In short, SGKL treatment induced the alteration of gut microbiota that led to changes in metabolite production, which caused the inactivation of the PI3K/Akt/mTOR pathway in the hippocampus and then inhibited the development of behaviors and inflammation. The regulation of gut microbiota in the PI3K/Akt/mTOR pathway may be the potential mechanism of SGKL between microglia and intestinal barrier. SGKL treatment improves the depression‐induced alteration of gut microbiota that stimulates the phosphorylation of the PI3K/Akt/mTOR pathway, which represses microglial activation through PI3K/Akt/mTOR pathway‐regulated inflammation in the hippocampus.

We demonstrated a novel mechanism of gut microbiota in causing depression; gut microbiota cause changes in phosphorylation of the PI3K/Akt/mTOR pathway, which mediates microglial activation and subsequent inflammation, eventually causing the development of depression. Importantly, SGKL treatment targets the PI3K/Akt/mTOR pathway in improving depression‐like behavior, microglial activation, and inflammation. Our investigation revealed the molecular mechanism of SGKL in gut microbiota‐associated depression and may be useful in promoting its clinical value in depression treatment.

## 5 LIMITATION

Gender plays a significant role in brain vessels, cerebral blood flow, brain metabolism, and animal behavioral deficits in CNS disorders,^51^, ^52^, ^53^, ^54^ which possibly affect the therapeutic effect of SGKL in depression. In the present study, we only explored the therapeutic mechanism of SGKL based on the gut–brain axis in male rats. In the present study, we only explored the therapeutic mechanism of SGKL based on the gut–brain axis in male rats. Actually, gender has been determined as a significant role affecting brain vessels, cerebral blood flow, brain metabolism, and animal behavioral deficits in CNS disorders, which possibly affect the therapeutic effect of SGKL in depression. We will design two experiments to investigate the role of gender during SGKL treatment. One experiment will contain the male rat group (*n* = 8) and female rat group (*n* = 8), both of which are exposed to chronic restraint stress and treated with SGKL. Another will include three groups: control without any stress, CRS exposed to chronic restraint stress, and SGKL treated with SGKL undergoing CRS modeling; in this experiment, each group contains eight mice in half genders. Also, it remains unclear how *Candidatus Arthromitus* is related to depression‐involved metabolites, which warrants further investigations based on fecal microbiota transplantation.

## CONFLICT OF INTEREST

None.

## ETHICAL APPROVAL

All animal works were conducted with the approval of the Ethics Committee of China‐Japan Friendship Hospital.

## Supporting information


Figure S1

Figure S2

Table S1

## Data Availability

The data sets used or analyzed during the current study are available from the corresponding author on reasonable request.
