# Supplementary figures and images for: Shugan granule contributes to the improvement of depression‐like behaviors in chronic restraint stress‐stimulated rats by altering gut microbiota
Source: CNS Neurosci Ther. 2022 Jun 17;28(9):1409–24. doi: 10.1111/cns.13881 (PMC9344086; doi:10.1111/cns.13881)

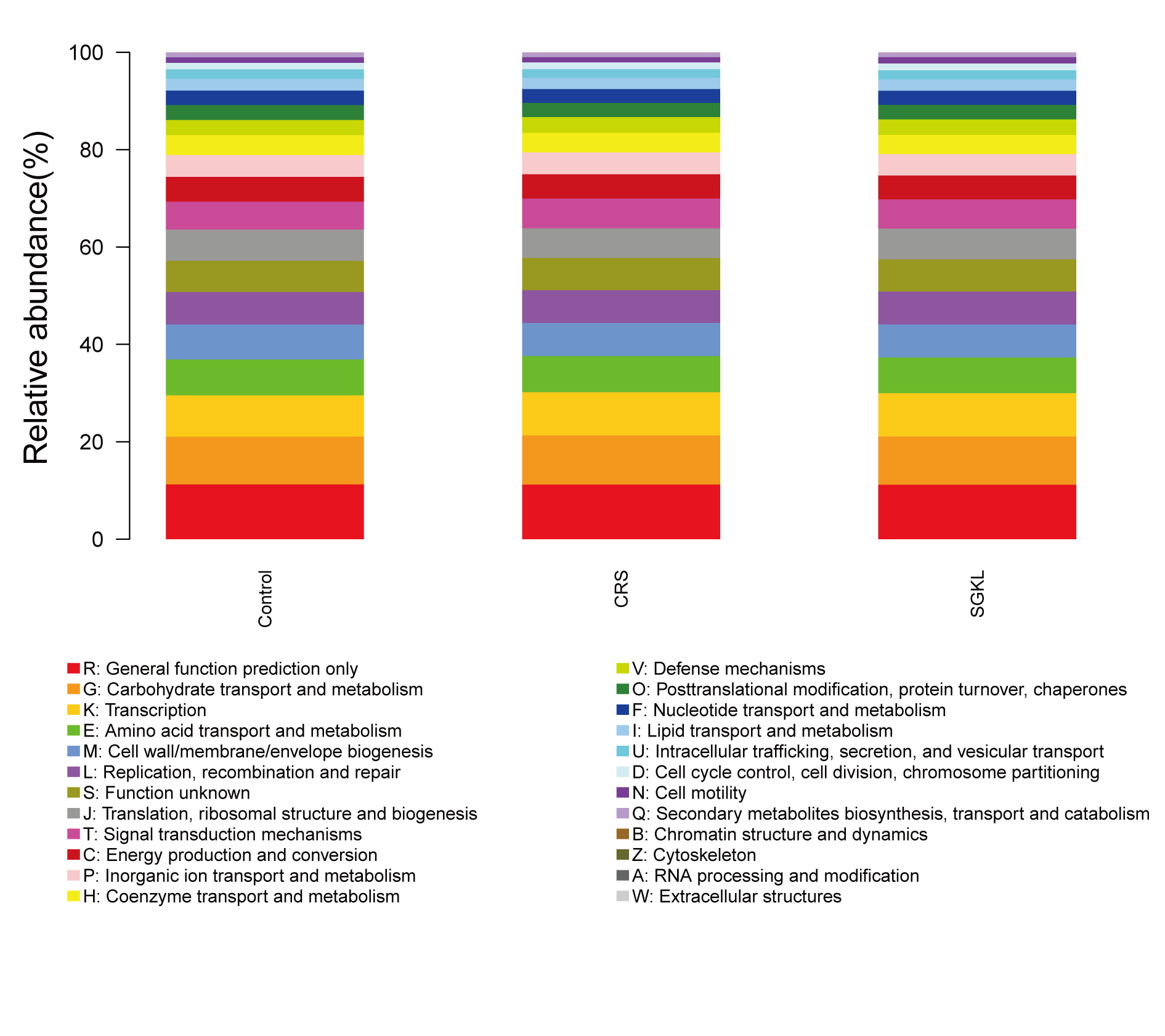

Supplement: Supplementary file 1 — Figure S1 [file CNS-28-1409-s003.jpg]

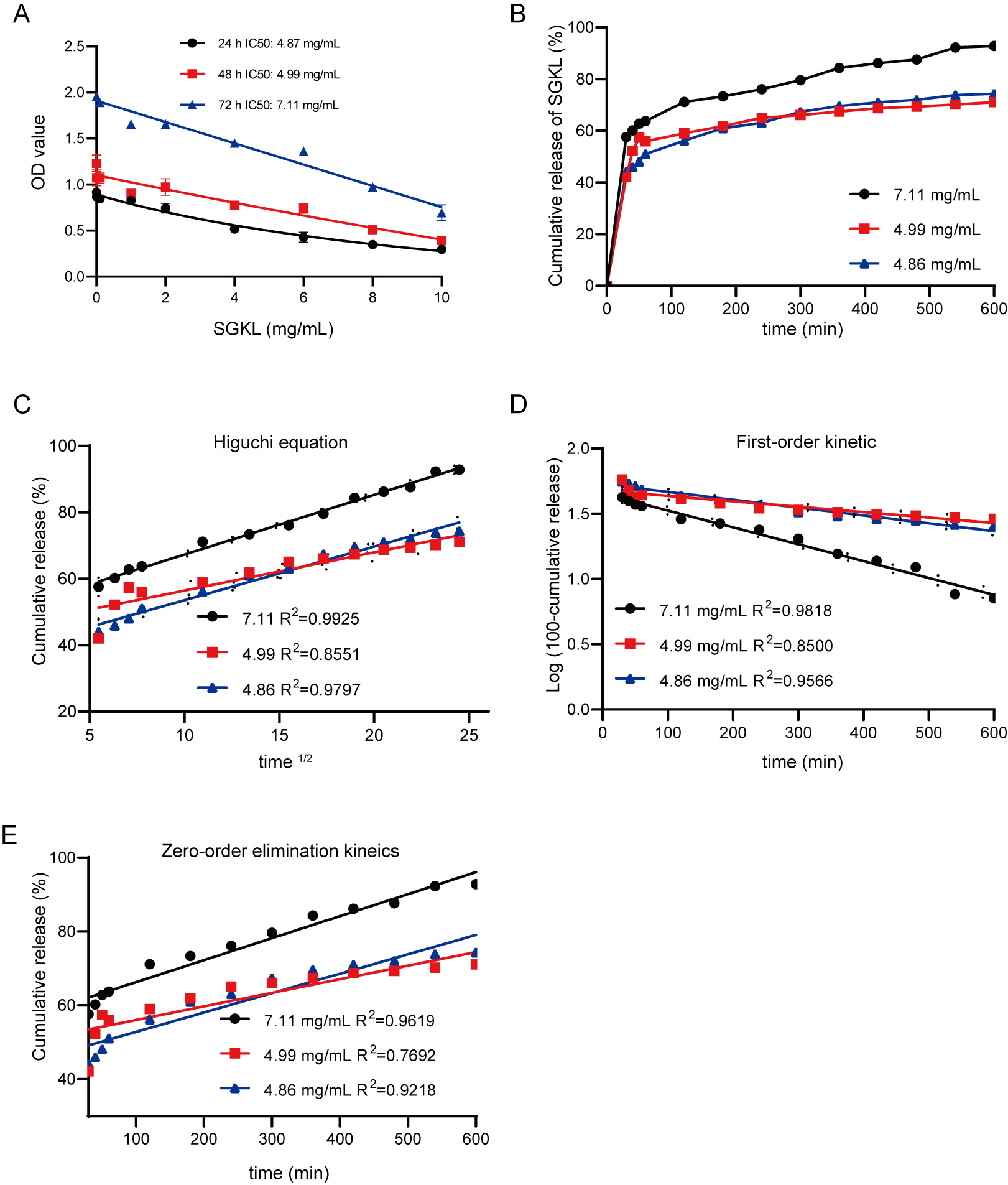

Supplement: Supplementary file 2 — Figure S2 [file CNS-28-1409-s002.jpg]
