# Supplementary material for: Shugan granule contributes to the improvement of depression‐like behaviors in chronic restraint stress‐stimulated rats by altering gut microbiota
Source: CNS Neurosci Ther. 2022 Jun 17;28(9):1409–24. doi: 10.1111/cns.13881 (PMC9344086; doi:10.1111/cns.13881)
Supplement: Supplementary file 3 — Table S1 [file CNS-28-1409-s001.docx]

**Table 1 Altered metabolites in SGKL group compared with CRS group**

| **Metabolite name** | **VIP** | **P-value** | **log2(FC)** |
| --- | --- | --- | --- |
| Ascorbic acid | 2.71073 | 0.02082 | -1.4389 |
| 3,4-dihydroxymandelic acid | 2.60622 | 0.01829 | -2.0076 |
| 4-hydroxyhippuric acid | 2.58182 | 0.00111 | -1.7784 |
| Pseudo uridine | 2.58113 | 0.02867 | 2.72794 |
| Glycerol-3-galactoside | 2.49082 | 0.01484 | -3.1574 |
| P-octopamine | 2.38823 | 0.03389 | 2.21167 |
| Daidzein | 2.29692 | 0.00265 | -1.7933 |
| Glycerol | 2.22276 | 0.00118 | 1.68642 |
| Saccharic acid | 2.12167 | 0.00281 | -1.7861 |
| D-myo-inositol 4-phosphate | 2.02867 | 0.03691 | 1.71244 |
| 5-hydroxyindoleacetic acid | 2.01216 | 0.00534 | -1.2309 |
| 3-deoxyhexitol | 1.97072 | 0.01417 | -1.7842 |
| Conduritol-beta-epoxide | 1.92562 | 0.00404 | -1.2579 |
| Glutamyl-valine | 1.92054 | 0.04315 | 2.4045 |
| Protocatechoic acid | 1.82 | 0.00788 | -1.1644 |
| 1-monopalmitin | 1.81388 | 0.00012 | 1.03825 |
| Uric acid | 1.8135 | 0.03587 | 1.74844 |
| Xylofuranose | 1.78911 | 0.00804 | -1.6731 |
| Nonadecanoic acid | 1.75426 | 0.00508 | 1.14623 |
| Uridine | 1.75421 | 0.01325 | -1.8981 |
| Cyclohexanecarboxylic acid | 1.74795 | 0.04197 | -0.8266 |
| Sophorose | 1.74222 | 0.00467 | -1.3447 |
| Xanthine | 1.7201 | 0.01837 | 1.24452 |
| Chenodeoxycholic acid | 1.71719 | 0.01329 | 1.17111 |
| Phytanic acid | 1.70403 | 0.0043 | 1.02205 |
| 3,4-dihydroxyhydrocinnamic acid | 1.69967 | 0.01927 | -1.1467 |
| Arabinofuranose | 1.68118 | 0.01315 | -1.801 |
| Xylonic acid | 1.67225 | 0.00138 | -1.0299 |
| Thymine | 1.65264 | 0.00851 | 1.16202 |
| Maltitol | 1.60813 | 0.03827 | 1.16013 |
| Citric acid | 1.59944 | 0.0004 | 0.84979 |
| 5-ethyl-1h-indole-2-carboxylic acid | 1.56998 | 0.00168 | -0.8191 |
| Chlorogenic acid | 1.54858 | 0.0215 | -1.1854 |
| Dihydrocholesterol | 1.51108 | 0.0149 | 1.00002 |
| Phytosphingosine | 1.50738 | 0.01403 | -1.8704 |
| 2'-deoxyguanosine | 1.49487 | 0.016 | 0.9237 |
| Aminomalonate | 1.47299 | 0.00321 | 0.79997 |
| Rhamnose | 1.45635 | 0.00547 | -1.5484 |
| Methanephosphonothioic acid | 1.45051 | 0.03117 | -0.8845 |
| Piceatannol | 1.4444 | 0.00093 | 0.78367 |
| Pectin | 1.43514 | 0.01549 | -1.5111 |
| Hypoxanthine | 1.43357 | 0.03937 | 0.96872 |
| Thymidine | 1.41263 | 0.01855 | 1.01137 |
| Uracil | 1.39111 | 0.00512 | 0.80434 |
| Alpha tocopherol | 1.36667 | 0.00503 | 0.69362 |
| 2,3-dihydroxybutanoic acid | 1.34144 | 0.0185 | -0.9308 |
| Beta-glutamic acid | 1.328 | 0.0242 | 0.86916 |
| Stigmasterol | 1.31118 | 0.00775 | -0.6462 |
| Lauric acid | 1.29602 | 0.01612 | -0.9282 |
| Tromethamine | 1.29169 | 0.03949 | -0.6393 |
| Benzoic acid | 1.27592 | 0.01336 | 0.7286 |
| 2-deoxyerythritol | 1.2733 | 0.00258 | -0.6098 |
| Pentadecanoic acid | 1.26479 | 0.00781 | 0.65098 |
| Pantothenic acid | 1.25767 | 0.03737 | 0.78178 |
| 3-hydroxymethylglutaric acid | 1.24587 | 0.02378 | -1.5688 |
| 2-hydroxy-3-methylbutyric acid | 1.23074 | 0.01489 | 0.66245 |
| Isomaltose | 1.2101 | 0.01957 | -1.6423 |
| Beta-mannosylglycerate | 1.20782 | 0.01976 | -1.639 |
| Cytosine | 1.17762 | 0.0496 | 1.00417 |
| Maleimide | 1.17345 | 0.00953 | -0.5337 |
| Triacontanol | 1.1651 | 0.02192 | -0.5897 |
| Gentisic acid | 1.1613 | 0.04466 | -0.8409 |
| 2-(4-hydroxyphenyl) ethanol | 1.15427 | 0.02508 | -0.6374 |
| N-acetyl-d-tryptophan | 1.15294 | 0.01766 | 0.54022 |
| Ethyl beta-d-galactofuranoside | 1.13154 | 0.04581 | 0.56025 |
| L-cysteine | 1.12357 | 0.0055 | -0.6283 |
| Palmitic acid | 1.11774 | 0.00556 | 0.48491 |
| Palatinitol | 1.07695 | 0.00799 | -0.5199 |
| 1,4-dihydroxy-2,6-dimethoxybenzene | 1.07324 | 0.01041 | -0.5022 |
| Glycocyamine | 1.04191 | 0.01262 | 0.44695 |
| N-methylglutamic acid | 1.03512 | 0.01827 | 0.50848 |
| Caffeic acid | 1.02735 | 0.00413 | 0.40836 |
| Malonic acid | 1.02207 | 0.03126 | -0.5211 |
| 5-hydroxyindole-2-carboxylic acid | 1.00033 | 0.0378 | -0.4496 |

Table 2 Altered metabolites in CRS group compared with control group

| **Metabolite name** | **VIP** | **P-value** | **log2(FC)** |
| --- | --- | --- | --- |
| 6-hydroxy-2-aminohexanoic acid | 2.946218489 | 0.000522586 | 5.595415908 |
| Triphenyl-phosphine imide | 2.875236311 | 0.018767925 | 6.714804145 |
| Acifluorfen | 2.865977279 | 0.018865053 | 6.683226294 |
| Normorphine | 2.810602276 | 0.000305975 | 5.227075488 |
| Uridine | 2.730727453 | 0.00159543 | 5.340510178 |
| 2-piperidinobenzonitrile | 2.728834328 | 9.63759E-08 | 4.352267311 |
| Pectin | 2.718962244 | 0.00068161 | 5.164941946 |
| Arabinofuranose | 2.655210022 | 0.001389426 | 5.013236875 |
| Estradiol | 2.588483807 | 0.000544677 | 4.647704702 |
| Rhamnose | 2.429447041 | 0.000248088 | 4.186473665 |
| 2-desoxy-pentos-3-ulose | 2.427776091 | 3.92342E-06 | 3.750389423 |
| Ascorbic acid | 2.392859414 | 0.010823005 | 1.868768339 |
| D-fructose-1-phosphate | 2.344298991 | 4.99998E-09 | 3.329436585 |
| 2-indolecarboxylic acid | 2.339335082 | 0.002730434 | 4.261349693 |
| Putreanine | 2.330462933 | 0.004321367 | 4.258355481 |
| Bumetanide | 2.320695367 | 0.00970533 | 4.344199305 |
| 5-methoxyindoleacetate | 2.313520485 | 0.00667215 | 4.091402066 |
| 4-hydroxyhippuric acid | 2.183853945 | 5.06082E-06 | 3.064689169 |
| 2,3-dihydroxy-2-butenedioic acid | 2.116222426 | 0.002445561 | 3.351208737 |
| 1-monopalmitin | 2.080085939 | 2.92639E-05 | 2.807672238 |
| Butorphanol | 2.072094911 | 0.015620191 | 3.527450227 |
| Xylofuranose | 2.059233511 | 0.001014859 | 3.251977484 |
| 3,5-dihydroxybenzoic acid | 1.886784763 | 0.021625857 | 2.916181594 |
| Resveratrol | 1.884023523 | 0.002003592 | 2.859280284 |
| Chlorogenic acid | 1.825423553 | 0.001350322 | 2.334735559 |
| Tocopherol acetate | 1.808407616 | 0.027317077 | 3.265713393 |
| Malate | 1.784374327 | 0.047540339 | -2.563035051 |
| 5-nonanol | 1.749414438 | 1.37156E-05 | 2.01630025 |
| L-aspartic acid | 1.733518368 | 0.01316267 | 2.547943448 |
| Maltitol | 1.728280251 | 0.000167962 | -2.156391355 |
| Creatinine | 1.70737043 | 0.010463209 | 2.734018143 |
| Phytosphingosine | 1.699285899 | 0.003350233 | 3.145521225 |
| 5-methoxytryptamine | 1.695824599 | 6.2928E-09 | -1.833360163 |
| 2,4-diaminobutyric acid | 1.691938235 | 0.001714565 | 2.252877245 |
| Protocatechoic acid | 1.689509647 | 8.6833E-05 | 2.028143397 |
| L-asparagine | 1.674750852 | 0.000668952 | 2.032732932 |
| 3-hydroxybenzoic acid | 1.670122159 | 0.016641899 | 2.472142117 |
| 3-methoxy-5-prop-2-en-1-yl-2-hydroxy-n-2-hydroxyethylbenzamide | 1.655032928 | 0.003974856 | 2.024242 |
| Isomaltose | 1.637934013 | 0.00346693 | 3.048758519 |
| 4-hydroxymandelic acid | 1.624553801 | 2.5276E-05 | -1.733937728 |
| Aminomalonate | 1.610714836 | 0.000105846 | -1.796752741 |
| 3-methylene-2-phenyl-1-hydroxy-cyclopentene | 1.592968499 | 0.000941683 | -1.872894895 |
| Methanephosphonothioic acid | 1.592321489 | 0.00029853 | 1.900590596 |
| 1,8-dihydroxynaphthalene | 1.588215925 | 0.00028083 | 2.055340117 |
| Pimaric acid | 1.582196364 | 0.002742573 | 2.049695694 |
| Beta-mannosylglycerate | 1.577035591 | 0.003737914 | 2.94254285 |
| N-acetyl-d-tryptophan | 1.575327518 | 4.46631E-06 | 1.638926665 |
| L-tryptophan | 1.569911759 | 0.000910309 | 2.084705428 |
| Boric acid | 1.543337342 | 4.2398E-07 | 1.549993021 |
| N-acetylornithine | 1.541683455 | 8.04797E-09 | -1.497019711 |
| Inosine-5'-monophosphate | 1.535207762 | 0.005767137 | 1.615350478 |
| Pyruvic acid | 1.497988105 | 0.001824334 | -1.86885292 |
| 2-aminoheptanedioic acid | 1.496731735 | 0.027733836 | 2.154331467 |
| P-octopamine | 1.484704671 | 0.007662216 | -2.044324685 |
| Erythronic acid | 1.480282001 | 0.000350482 | 1.745550648 |
| Citric acid | 1.463375477 | 0.000350944 | 1.522059648 |
| 3,4-dihydroxyhydrocinnamic acid | 1.458703625 | 0.001579365 | 1.775002334 |
| Acebutolol | 1.446795341 | 1.57904E-05 | 1.441545748 |
| Dehydroascorbic acid | 1.430006837 | 0.000817474 | 1.666887963 |
| 2,3-dihydroxy-acrylic acid | 1.419091735 | 0.001619152 | 1.750303093 |
| 3-aminoisobutanoic acid | 1.415958974 | 0.006808279 | 1.862403847 |
| L-cysteine | 1.414516423 | 4.02876E-05 | 1.454561691 |
| 2,3-dihydroxybutanoic acid | 1.394326035 | 0.032752808 | 1.280084102 |
| Putrescine | 1.392893695 | 5.01892E-10 | -1.2184166 |
| Tryptamine | 1.389771377 | 0.004238203 | 1.373457028 |
| 3,4-dihydroxymandelic acid | 1.371392544 | 0.023274514 | 1.745770314 |
| Galacturonic acid | 1.357597088 | 1.28555E-05 | 1.262626827 |
| Alpha tocopherol | 1.349119426 | 0.000379519 | -1.330448813 |
| 2,5-piperazinedione | 1.3363084 | 0.000720737 | 1.307907765 |
| O-phosphoserine | 1.334357905 | 0.008496332 | -1.523078221 |
| Gallic acid | 1.333510045 | 0.003910843 | 1.808156602 |
| L-glutamic acid | 1.322345139 | 0.000249114 | 1.337713705 |
| 3-hydroxyphenylacetic acid | 1.2975276 | 0.002392309 | -1.275357214 |
| Malonic acid | 1.295641007 | 0.000308314 | 1.330833368 |
| Galactinol | 1.289685019 | 0.004348801 | -1.396251199 |
| 3-hydroxymethylglutaric acid | 1.287308555 | 0.00766283 | 2.18096634 |
| Dimethyluric acid | 1.274902479 | 0.002135626 | 1.311711195 |
| Saccharopine | 1.273789519 | 0.012209377 | -1.200416525 |
| 5-aminovaleric acid | 1.253256148 | 0.013944878 | 1.301323642 |
| 2-ketoisovaleric acid | 1.251389292 | 0.007433519 | -1.377739904 |
| L-methionine | 1.251133226 | 0.013181249 | 1.127797398 |
| Ribonic acid | 1.248563455 | 0.008735208 | 1.289140213 |
| Coniferin | 1.243732877 | 0.024563819 | -2.080676841 |
| Isoproterenol | 1.236654196 | 0.028260678 | 1.355837718 |
| Hexuronic acid | 1.235760768 | 0.000548891 | -1.115302388 |
| Guanosine | 1.234704731 | 0.003164713 | -1.163207052 |
| 1,4-dihydroxy-2,6-dimethoxybenzene | 1.231797844 | 1.7554E-05 | 1.043329602 |
| Sophorose | 1.229795434 | 0.004016893 | 1.409787743 |
| 4-hydroxyamphetamine | 1.218618727 | 0.003910406 | 1.118471396 |
| N-acetyl-d-mannosamine | 1.210348193 | 0.001023796 | -1.180509674 |
| Mannitol | 1.210004652 | 3.60825E-08 | -0.940523914 |
| L-cystathionine | 1.199572896 | 0.001480141 | 1.193625193 |
| Methyl beta-d-glucopyranoside | 1.19770846 | 0.01980531 | -1.409649615 |
| 2,3-dihydro-8-methoxyfuro(2,3-b)quinoline | 1.190717791 | 2.16027E-10 | -0.893223566 |
| 4-hydroxybenzeneacetic acid | 1.181300159 | 0.046199329 | 1.299801193 |
| Piceatannol | 1.146182123 | 0.000453976 | 1.00974877 |
| Pentitol | 1.140353439 | 3.38833E-05 | 0.953404766 |
| Glycyl proline | 1.135928638 | 0.002674385 | 1.07995115 |
| Deoxyribose | 1.135184765 | 0.013323907 | -1.315331594 |
| Mannofuranuronic acid | 1.13052572 | 0.000413425 | 0.972622048 |
| 2-methyl-propanetriol | 1.112483799 | 0.02750294 | -1.487177174 |
| L-threonic acid | 1.109902839 | 0.001122774 | 1.089209301 |
| 2-o-methyl-l-ascorbic acid | 1.10483548 | 0.027567612 | 1.585953793 |
| Leucine | 1.104227111 | 0.021800313 | 0.827087313 |
| Nonanoic acid | 1.091194812 | 0.047417473 | 0.987662259 |
| Deoxycholic acid | 1.080316631 | 0.012624407 | 1.164484699 |
| Oxoproline | 1.07580452 | 0.000266721 | 0.881547733 |
| Melibiose | 1.041309111 | 0.049196561 | 1.531707743 |
| Catechollactate | 1.039417742 | 0.023946826 | 1.211622907 |
| Pipecolic acid | 1.031708372 | 2.21216E-05 | 0.76247293 |
| Dl-dopa | 1.016308067 | 0.048966165 | 0.920202018 |
| Ergosterol | 1.009545695 | 0.001692942 | 0.817619126 |
| Chenodeoxycholic acid | 1.003846243 | 0.001602184 | 0.880299803 |
| L-arabitol | 1.001977028 | 0.047390426 | 0.79896204 |

Figure S1 Functional inference of COG for the altered gut microbiota based on Phylogenetic Investigation of Communities by Reconstruction of Unobserved States (PICRUSt) and LEfSe. We showed the relative abundance of COG categories among the three group.

Figure S2 Selection of SG concentration at cellular level

A, dose-cell viability curve by MTT assay. B, release curve of SG in vitro. C, Higuchi equation fitting of release curve. D, first-order kinetic fitting of release curve. E, zero-order kinetic fitting of release curve.
